# Patient Breathing Motion and Delivery Specifics Influencing the Robustness of a Proton Pancreas Irradiation

**DOI:** 10.3390/cancers15092550

**Published:** 2023-04-29

**Authors:** Barbara Knäusl, Franciska Lebbink, Piero Fossati, Erik Engwall, Dietmar Georg, Markus Stock

**Affiliations:** 1 Department of Radiation Oncology, Medical University of Vienna, 1090 Vienna, Austria; 2 MedAustron Ion Therapy Centre, Medical Physics, 2700 Wiener Neustadt, Austria; 3 Division Medical Physics, Karl Landsteiner University of Health Sciences, 2700 Wiener Neustadt, Austria; 4 RaySearch Laboratories, 10430 Stockholm, Sweden

**Keywords:** 4D, proton therapy, dose calculations, pancreatic cancer, interplay

## Abstract

**Simple Summary:**

The study retrospectively analysed the clinical proton treatment strategy for pancreas patients with small moving tumours. Seventeen hypofractionated proton treatment plans were analysed based on 4D dose calculations, which gives insight into the interplay of beam and organ motion. The results showed that the synchrotron-based dose delivery employing horizontal and vertical beams and robust optimisation was robust against intra-fractional movements up to 3.7 mm. However, outliers were observed in some patients, indicating the need for continuous monitoring during clinical practice to identify patient cases with more significant deviations. The research serves as a basis for future treatment strategies for patients with larger motion amplitudes and the transition towards carbon ion treatments.

**Abstract:**

Motion compensation strategies in particle therapy depend on the anatomy, motion amplitude and underlying beam delivery technology. This retrospective study on pancreas patients with small moving tumours analysed existing treatment concepts and serves as a basis for future treatment strategies for patients with larger motion amplitudes as well as the transition towards carbon ion treatments. The dose distributions of 17 hypofractionated proton treatment plans were analysed using 4D dose tracking (4DDT). The recalculation of clinical treatment plans employing robust optimisation for mitigating different organ fillings was performed on phased-based 4D computed tomography (4DCT) data considering the accelerator (pulsed scanned pencil beams delivered by a synchrotron) and the breathing-time structure. The analysis confirmed the robustness of the included treatment plans concerning the interplay of beam and organ motion. The median deterioration of *D*_50%_ (Δ*D*_50%_) for the clinical target volume (CTV) and the planning target volume (PTV) was below 2%, while the only outlier was observed for Δ*D*_98%_ with −35.1%. The average gamma pass rate over all treatment plans (2%/ 2 mm) was 88.8% ± 8.3, while treatment plans for motion amplitudes larger than 1 mm performed worse. For organs at risk (OARs), the median Δ*D*_2%_ was below 3%, but for single patients, essential changes, e.g., up to 160% for the stomach were observed. The hypofractionated proton treatment for pancreas patients based on robust treatment plan optimisation and 2 to 4 horizontal and vertical beams showed to be robust against intra-fractional movements up to 3.7 mm. It could be demonstrated that the patient’s orientation did not influence the motion sensitivity. The identified outliers showed the need for continuous 4DDT calculations in clinical practice to identify patient cases with more significant deviations.

## 1. Introduction

Pancreatic cancer is known to be one of the most radioresistant tumour types and therefore extremely difficult to treat. Charged particle therapy has a large potential to play a major role in improving oncologic outcomes [1]. Due to the distinct physical properties of protons and carbon ions, i.e., the sharp dose fall-off (Bragg peak) and the increased relative biological effectiveness (RBE), adjacent organs at risk (OARs) can be spared while the dose to the tumour can be escalated compared to photon therapy [2,3]. For particle therapy, the inherent range uncertainties due to inter-fractional anatomical changes, patient positioning, or intra-fractional motion, like breathing have a higher impact on the dosimetric accuracy compared to photon radiotherapy [4]. Especially in combination with varying beam delivery dynamics intra-fractional anatomical variations showed to affect the dose distribution in terms of homogeneity, the target coverage and dose to the surrounding tissue [5]. The simultaneous movement of the beam or machine parts (e.g., leaves and gantry), and the organ/target motion caused by breathing may cause hot and cold spots for all treatment techniques. This so-called interplay effect is most pronounced for pencil beam scanning [4,6,7]. Changes to existing treatment schemes, e.g., hypofractionation or using different particle types, require quantifying the interplay effect independently of the pathology. The same applies to the inclusion of larger tumour amplitudes.

Recent preparatory time-resolved measurements with an anthropomorphic breathing phantom [8,9] demonstrated that no motion mitigation was needed for protons for breathing motions within 1 cm, while carbon ions were more sensitive towards intra-fractional motion effects [10]. Further, experimental validation of different 4D dose tracking (4DDT) methods showed excellent results serving as a basis for the use in the clinical workflow [11,12,13].

Complementary to the phantom studies, the presented retrospective study on clinical data sets addresses the impact of a combination of the most relevant intra-fractional variations. The interplay effect, that might occur during synchrotron-based proton irradiation for pancreas patients was quantified employing the validated log-file based 4DDT framework for protons [13].

## 2. Materials and Methods

### 2.1. Patient Data, Imaging and Treatment Workflow

Twelve pancreatic patients (age range: 48 to 80, 7 male, 5 female) treated at the fixed horizontal and vertical beam lines of the MedAustron Ion Therapy centre (MedAustron) with definitive proton radiotherapy within the registry trial (trial number GS1-EK-4/350-2015) were included in the retrospective 4DDT analysis. The clinical target volume (CTV) size ranged from 115.5 cm^3^ to 332.9 cm^3^ and a 5 mm isotropic planning target volume (PTV) margin was applied. The patient collective encompassed all pancreatic patients with high quality 4D computed tomography (4DCT) examinations which were treated between 2019 and 2021. In this period patients were immobilized including abdominal compression via a mask to minimize motion and hence could be classified as small movers.

**Figure 1:**
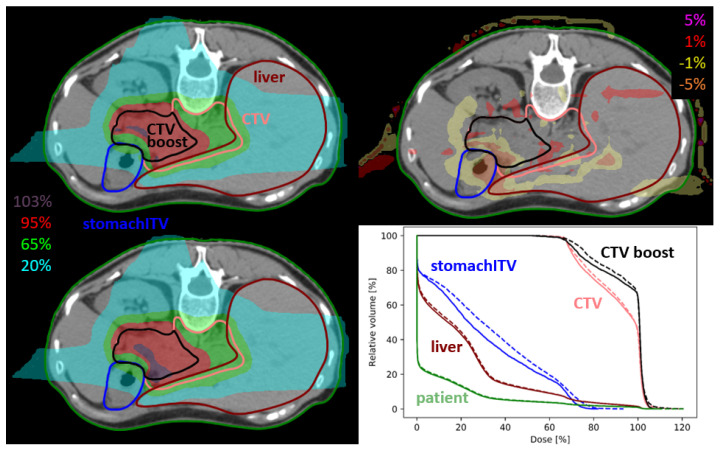
Dose distribution of static plan (**upper left**), 4DDT dose (**lower left**), dose difference (**upper right**) and dose volume histogram (DVH) curves (**lower right**) of pan13. The solid line in the DVH represents the static dose and the dashed line is the 4DDT dose for the CTVs, stomach and liver.

Patients were treated on five days, either with a single fraction per day or twice per day, as summarised in Table 1. For the patients treated twice a day the prescribed fraction dose was split in two, which resembles a simple rescanning approach. The mean RBE-weighted prescribed dose (based on a constant biological weighting factor of 1.1) varied between 35 to 40 Gy(RBE) for PTV_boost_ and 25 Gy(RBE) for the PTV. The CTVs, PTVs as well as all necessary OARs as liver, stomach, kidneys, and spinal cord were defined on the computed tomography (CT) scan as illustrated in Figure 1, which further served for treatment plan creation.

Planning CT and 4DCT images were acquired with the Brilliance CT Big Bore scanner (Philips, The Netherlands) employing the clinical acquisition protocol for the abdomen ( 120 kV, 300 mAs, slice thickness 2 mm or 3 mm). For a reproducible position during imaging and every treatment fraction, a vacuum mattress in combination with mould care devices and a thermoplastic mask for abdominal compression was used. All patients were immobilised in HFP position except for three cases. Phase-based 4DCT scans were recorded with eight respiratory phases employing the Sentinel surface scanner (C-RAD, Sweden). All 4DCT scans were acquired subsequent to the planning CT keeping the patient in the identical position.

The treatment rooms are equipped with a couch-mounted imaging device (ImagingRing™, medPhoton GmbH, Austria) [14], a tracking camera, and a patient positioning system (Exacure, BEC GmbH, Germany) [15] as well as a Catalyst surface scanner system (C-RAD, Sweden). Patient positioning was verified using two orthogonal kV images and surface imaging during every fraction.

Clinical proton treatment plans were created on the planning CT in RayStation v7.99 (RaySearch Laboratories AB, Stockholm, Sweden) using the Hounsfield units (HU) to stopping-power calibration curve commissioned for the clinical CT protocol for abdominal imaging [14]. The dose was computed (Monte Carlo (MC) v4.3) on a 2 mm × 2 mm × 2 mm calculation grid. Plan optimisation was performed on the PTV and according to the clinical constraints defined for each patient individually. Robust optimisation considering 3% range and 3 mm setup uncertainties was added to the cost functions for the CTV and close-by sensitive OARs to assure target coverage and sparing of those OARs, i.e., liver and spinal cord.

**Figure A1:**
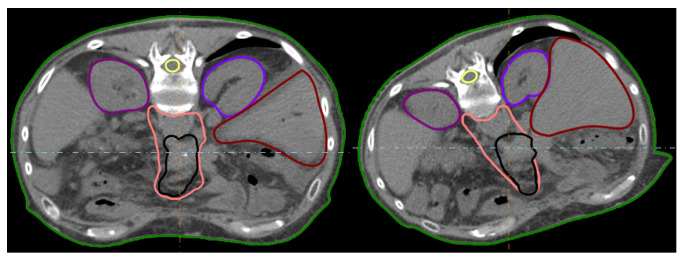
Illustration of the two patient orientations during immobilisation (straight and rotated) exemplary for pan12.

A beam configuration employing vertical and horizontal directions was used for all treatment plans. The horizontal beam was combined with a couch rotation of up to 25° depending on the tumour position. To increase the possible beam incidence directions an additional planning CT, with the patient in a rotated position, was acquired for several patients, further called ‘rotated CT’ (exemplarily shown in Figure A1). This resulted in 18 treatment plans from twelve patients, while the effect of the simple beam arrangement and different orientations was especially considered in the evaluation. One treatment plan could not be evaluated due to a large number of spots that was not supported in this version of the 4DDT tool, so in total 17 treatment plans remained for evaluation. For evaluation purposes treatment plans were grouped by the corresponding motion amplitude retrieved from the 4DCT as listed in Table 2: motion amplitudes >1 mm (8 plans on 8 CTs) and non-movers (<1 mm) (9 plans on 9 CTs).

Hybrid deformable image registrations (DIRs) implemented in the treatment planning system (TPS) were applied between the different phases of the 4DCT and the planning CT [16]. The deformable registration between the different phases of the 4DCT was additionally used to extract the breathing amplitude. A point of interest (POI) on the patient’s surface was selected with the same cranio-caudal and left-right coordinates as the centre of the PTV. The geometric POI statistics between the different phases defined the maximum amplitude in the anterior-posterior direction.

All patients were treated with scanned pulsed quasi-discrete spot scanned proton beams delivered by a synchrotron. The maximal spill length was 5 s. During the period of this study (2019–2021) the energy switching time of the accelerator was decreased from 4 s to 2 s.

### 2.2. 4D Dose Tracking Framework

**Figure 2:**
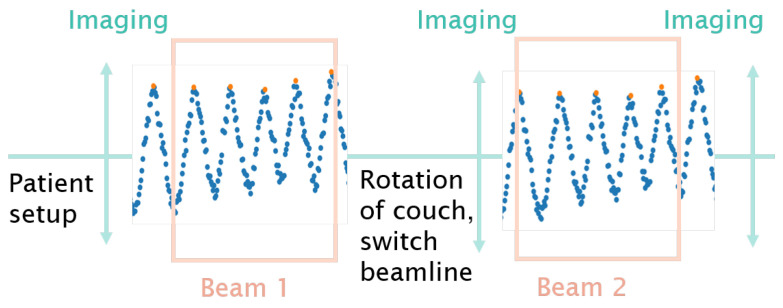
Schematic illustration of the workflow: patient positioning, in-room image acquisition, beam application and potential intermediate and exit imaging. The surface scanner acquisition was interrupted during couch movements caused by a change of the couch positioning, switch between horizontal and vertical beamline or imaging. The start of delivering beam#1 was assumed to be at the 0% breathing phase, while the 4DDT for beam#2 just continued taking the given treatment timeline into account.

4D dose tracking (4DDT) is the recalculation of the spots on the different 4DCT phases based on an external breathing signal while taking the dose delivery time structure into account. For the patient treatments included in this study gated beam delivery was not available. Therefore the spot delivery was not synchronised with the breathing signal during irradiation. It was assumed that the irradiation started at the 0% breathing phase, illustrated in the workflow diagram in Figure 2.

A tool for 4DDT was implemented in the treatment delivery module of the research version of RayStation 10 A (v10.0.110) and used in this study as follows:

- Distribute spots over different phases of the 4DCT for each fraction employing
  - A treatment record log file;
  - A breathing signal;
  - A map between breathing amplitude to phase;
  - Deformable image registration (DIR) between the different phases of the 4DCT and the planning CT.
- Compute dose on the different phases based on the determined spot distribution.
- Map doses to the reference phase, i.e., planning CT through DIR and accumulate the mapped doses
- Accumulate the 4DDT dose of each fraction to get the full treatment course dose

The accelerator log files, which were acquired during every single fraction contained the following delivery information on a spot-specific basis: spot position and energy, the number of particles delivered at the defined position, as well as the start time of every spot. From that information, spot timings were extracted for every single fraction of each patient summing up in 95 different fraction doses contributing to the analysis.

Due to the limited availability of real-time breathing data from the Catalyst in-room surface scanner, the breathing period was extracted from the 4DCT for most cases. The Sentinel surface scanner (C-Rad, Sweden) provided the breathing signal during 4DCT acquisition. The breathing curve was extracted by combining the Digital Imaging and Communications in Medicine (DICOM) information related to acquisition time (DICOM tag = ‘ContentTime’) and breathing phase (DICOM tag = ‘SeriesDescription’). To simulate a realistic daily variation, the breathing periods were shuffled and repeated to match the length of each treatment fraction and to include varying breathing patterns. Due to the use of a phase-based 4DCT, without the correlation to the respective amplitude, the breathing periods were divided into eight equal phases. The combination of the different breathing motion patterns and the daily delivery time structure simulated a realistic interplay effect taking into account the given fractionation scheme.

### 2.3. Rescanning

To determine the potential improvements of rescanning, log files were created for eight plans to compare the clinical rescanned plan (2 fractions a day) with a simulated plan with a two-fold fraction dose without rescanning. Log files were generated by doubling the number of particles per spot and taking into account the correct accelerator time structure. All further steps related to the 4DDT as described in Section 2.2 were performed in the same way as for the original treatment plans employing the log files and breathing amplitudes of the first fraction of each particular day.

### 2.4. Evaluation and Statistics

DVH parameters (*D*_2%_, *D*_98%_, *D*_50%_ and *V*_95%_) were extracted for the target volumes, i.e., PTV and CTV. The deterioration of DVH parameters observed with 4DDT, namely Δ*D*_50%_, Δ*D*_98%_ and Δ*D*_2%_ was calculated as follows: ΔD = ($D_{4\mathrm{DDT}}-D_{\mathrm{static}}$)/$D_{\mathrm{static}}$. The median over all the deteriorations of all patients was calculated. The homogeneity index (HI) was defined as (*D*_2%_ − *D*_98%_)/*D*_50%_ [17]. For selected OARs, i.e., liver, kidney, stomach, and spinal cord general DVH parameters were evaluated in addition to clinical dose constraints. For the liver, *D*_700cc_ was selected according to the Quantec report as a surrogate for adequate liver function with a limit of 15 Gy(RBE) [18]. OARs were excluded from the evaluation if $D_{2\%}$ was smaller than 1 Gy(RBE). All target and OAR parameters were evaluated by calculating the median of all treatment plans and reporting them with the respective range. Gamma pass rates were calculated in Python by comparing the 4DDT with the original plan with a 2%/2 mm criteria, once considering all dose values above a lower dose cutoff of 20%, and once only in the PTV. An unequal variance t-test, Welch’s t-test, had been performed (significance level *p* < 0.05) to compare the influence of different breathing amplitudes, CT orientations and PTV sizes on each other.

## 3. Results

**Figure 3:**
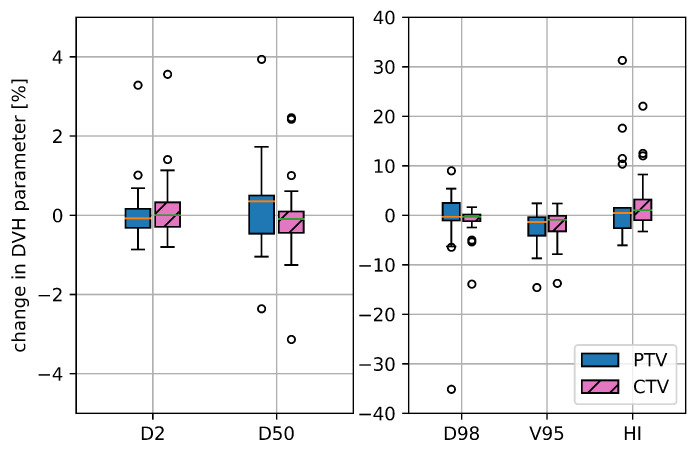
Deterioration for selected target DVH parameters for the initial CTV and PTV, namely *D*_2%_, *D*_50%_, *D*_98%_, *V*_95%_ and HI comparing the static dose distribution with the one retrieved from the 4DDT for all 17 treatment plans.

For the initial PTV 4DDT revealed a median deterioration of the static *D*_50%_ by 0.35% and of *D*_2%_ by −0.08% without remarkable outliers. Median PTV and CTV Δ*D*_98%_ decreased by approximately −0.30%, while the range of deterioration was essentially higher for the PTV (Δ*D*_98%_ ranging from −35.1 to 9.0%) than for the CTV (Δ*D*_98%_ ranging from −13.9 to 1.6%) as shown in Figure 3. The median HI increase was 0.44% (−6.1 to 31.3%) for the PTV and 1.04% (−3.3 to 22.0%) for the CTV. Median Δ*V*_95%_ was comparable for PTV and CTV with −1.36% and −0.93%, respectively. For the CTV_boost_ and PTV_boost_ comparable results were seen for considered dosimetric parameters, while all further evaluation of target parameters concentrated on the initial CTV and PTV. An exemplary dose distribution is depicted in Figure 1.

The evaluation of the target DVH parameters grouped according to the patient’s motion amplitude revealed that motion amplitudes > 1 mm lead to a higher variability for the DVH parameters. This was pronounced for the target, especially for the HI and the *D*_98%_, but the effect was not significant (*p* > 0.05). Δ*D*_98%_ was 0.05% (−3.8 to 3.3%) for amplitudes smaller than 1 mm and increased to −0.7% (−35.1 to 9.0%) for the bigger motion amplitudes. For *V*_95%_ the variation was similar for the two groups.

Assessing the effect of different patient orientations during positioning visible on the CT revealed a larger variation in the HI for the ‘rotated CTs’, while for *D*_98%_ and *V*_95%_ almost no difference was observed in between different orientations. The deterioration of the median HI was 0.44% for the ‘straight CTs’ and -0.20% for the ‘rotated CTs’ (*p* = 0.3). The gamma analysis showed a small non-significant difference between the *’rotated’* (87.6% ± 9.8) and the ‘straight CTs’ (89.4% ± 8.3).

Averaged over all treatment plans, the gamma pass rate (2%/2 mm) for the PTV region was 88.8% ± 8.3 (Table 2). Correlating the gamma pass rate to the breathing amplitude, the PTV region showed a gamma pass rate of 92.4% for breathing motions <1 mm, while this decreased to 84.7% for motions >1 mm (*p* = 0.07). Additionally, the correlation between the gamma pass rate and the PTV size was investigated. For PTV volumes larger than >350 cm^3^ a higher gamma pass rate was observed without revealing a significant difference compared to smaller volumes.

In 14 cases the static and 4DDT *D*_2%_ of the liver was above 1 Gy(RBE). The median $\Delta D_{2\%}$ comparing static and 4DDT was −0.58%. $D_{700\mathrm{cc}}$ of the liver was below 5 Gy(RBE) for all treatment plans. The median $\Delta D_{700\mathrm{cc}}$ averaged over the 14 included cases was 0%, ranging from −17.6 to 33.3%.

For the stomach the static $D_{2\%}$ values varied between 0 to 25.2 Gy(RBE) for the included cases. The median $\Delta D_{2\%}$ of the stomach was 1.5% (−6 to 160%). For patient pan4 $D_{2\%}$ increased from 0.26 to 10 Gy(RBE) (excluded from the median due to the 1 Gy(RBE) limit) for plan1 (‘straight CT’) and from 1.31 to 3.41 Gy(RBE) (change of 160%) for plan2 (‘rotated CT’), while the stomach moved into the beam path during breathing for both plans.

For patient pan16 the volume of the lung changed from 586 ${\mathrm{cm}}^{3}$ for the 4DCT 0% phase to 214 ${\mathrm{cm}}^{3}$ for the 4DCT 50% phase, which resulted in a decreased lung $D_{2\%}$ of the 4DDT by 1.5 Gy(RBE). For all other patients, the dose to lung dose varied within 1.0 Gy(RBE).

The median $\Delta D_{2\%}$ of the ipsilateral kidney was 0.16% due to motion (−9.6 to 4.6%), while the median $\Delta D_{2\%}$ of the contra-lateral kidney was −1.3% (−3.5 to 8.3%). Correlating the motion amplitude to $\Delta D_{2\%}$ for the ipsilateral kidney, showed a median significant (*p* = 0.004) change from 0.87% for the small motions to −2.75% for the bigger motions.

**Figure 4:**
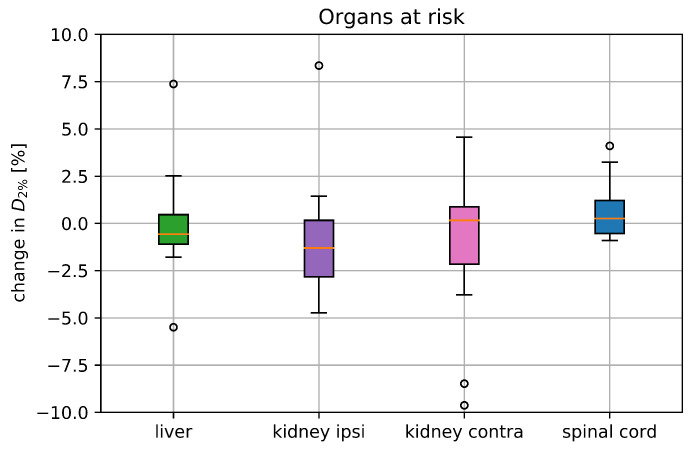
Deterioration of $D_{2\%}$ ($\Delta D_{2\%}$) for the liver, both kidneys and the spinal cord including all 17 treatment plans comparing the static dose distribution with the one retrieved from the 4DDT; parameters where the static $D_{2\%}$ was below 1 Gy(RBE) were excluded from the analysis. The boxplots show the median and the quartile values, where the circles represent the outliers.

The static spinal cord $D_{2\%}$ ranged from 0 to 13.8 Gy(RBE). $\Delta D_{2\%}$ was below 4.1% for all cases, shown together with the $\Delta D_{2\%}$ values of the other organs in Figure 4.

**Figure 5:**
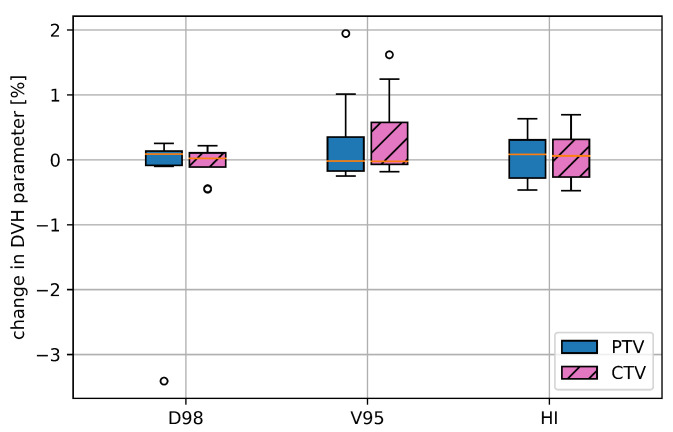
Deterioration of selected DVH parameters for the CTV and the PTV, namely, $D_{98\%}$, $V_{95\%}$ and HI comparing the 4DDT results of eight rescanned with the non-rescanned treatment plans.

The 4DDT analysis of the rescanned plans (as defined in Section 2.3) versus the simulated non-rescanned plans showed a median $\Delta D_{2\%}$ of −0.02% and $\Delta D_{50\%}$ of 0% without any outliers. The median $\Delta D_{98\%}$, $\Delta V_{95\%}$ and ΔHI was below 0.1% for the PTV and for CTV (shown in Figure 5). For patient pan7 (plan2) rescanning decreased $\Delta D_{98\%}$ between static and 4DDT from 12.8 to 9.0%, while for the other patients, the difference was within 0.5%.

Averaged over all cases the gamma pass rate for the PTV region was almost identical for rescanning (88.5% ± 9.5) and non-rescanning (88.9% ± 8.9).

## 4. Discussion

Clinical treatment plans for patients suffering from pancreatic cancer were retrospectively analysed concerning motion effects on the target coverage and OAR sparing. All included patients were classified as small movers (maximum motion amplitude 3.7 mm) without the need for motion mitigation. The robustly optimised clinically applied treatment plans were analysed fraction-wise based on the breathing information and the machine-specific delivery time structure.

Even the motion amplitudes of the included patients might not require motion mitigation techniques, such as gating or rescanning, an analysis on a centre-specific basis is necessary before considering larger motion amplitudes [9,10,19,20]. The evaluation of the data is following recent recommendations for real-time intrafactional motion management in particle therapy and quantifies the interplay effect on the planned dose distribution on a patient-specific basis [5,21].

The 4DDT covering the whole treatment period was based on the 4DCT which was acquired on the same day as the initial planning CT. This way the 4DDT analysis purely focused on the effect of motion while anatomical changes were not considered. While organ filling and movement could not be included in the analysis due to the lack of follow-up imaging data, it was considered during robust optimisation on several CTs with simulated varying filling status. The inclusion of an additional 4DCT or fraction-specific imaging information would complete the analysis but also adds additional DIR uncertainties, one of the major challenges during 4DDT [22,23,24]. Since most of the included patients were treated on five consecutive days, the DIR inaccuracies were reduced to a minimum by using only the planning CT and 4DCT on the same day before treatment. To further reduce the uncertainties of the DIR, to enable robust optimisation on the 4DCT and to optimize the workflow, it is recommended to use one phase of the 4DCT for planning. Anyhow, to achieve sufficient image quality requires a 4DCT acquisition protocol optimised for that purpose or the inclusion of 4D magnetic resonance imaging (MRI) information accounting for motion irregularities [25,26]. Motion irregularities or possible drifts were not considered in this study justified by the small breathing amplitude for the included patients.

The available infrastructure did not yet allow advanced methods like layer or spot rescanning. For some of the clinical treatment plans, the dose was simply divided into two equal fractions and irradiated directly after each other extending the total in-room time per day essentially. The results showed that the effect of this simple rescanning method was limited as depicted in Figure 5, which was also observed in a cyclotron-based study by Engwall et al. [27]. Even though the accelerator infrastructure was different, the combination of specific plan configurations, motion patterns and the delivery time structure could be the reason.

In a clinical setup, the 4DDT method presented in this study could be used to determine the accumulated dose on a daily basis during therapy and serve as a decision tool for plan adaptation. Although the breathing data from the 4DCT was used for most cases, the breathing patterns were varied by shuffling the breathing periods (mean breathing period shown in Table 2) for every considered fraction. By including the log files of every treatment session, the dose rate variation over the different fractions was taken into account (mean and standard deviation of the dose rate are shown in Table 1). The combination of the variation of the dose rate and the shuffled breathing patterns simulated the impact of the interplay effect by fractionation. Since the daily changes in the breathing patterns, available from the surface scanner (Table 1) was small, the presented evaluation approach can be considered to fulfil clinical requirements. Still, to reduce the uncertainties to a minimum, motion monitoring data from every fraction is recommended. Using the breathing pattern from the 4DCT in combination with log files acquired during patient-specific quality assurance (QA) before treatment starts could further serve as a prospective tool for predicting the worst-case scenario.

For pancreatic patients, Batista et al. [3] reported a deterioration of $V_{95\%}$ of the CTV of up to −28.0%, while our results showed a maximal $\Delta V_{95\%}$ of −13.7% The difference between the two patient collectives was mainly the motion amplitude, which was almost twice as large in the study by Batista et al. [3]. Similar results were reported by Ribeiro et al. [28] for lung and oesophagal cancer patients. Even large motion amplitudes were considered in their study (5.7 mm to 9.0 mm) *V*_95%_ of the CTV was above 98% for 80% of the patients with an outlier of 88% for one oesophagus patient. For the treatment plans analysed in our study CTV *V*_95%_ preservation was slightly worse even though the motion amplitudes were smaller. A major reason for that could be the better target coverage in the static plan reported by Ribeiro et al. [28], which might come along with a shallower dose fall-off towards the adjacent OARs.

Meijers [29] and Lim et al. [30] reported no clinically relevant loss of target dose homogeneity in the fraction-wise reconstructed 4D dose distributions for NSCLC and lymphoma patients as well as paediatric Neuroblastoma patients, respectively. In those studies, small motion amplitude [29], compensation by rescanning and target location [30] might have reduced the effect of motion on the target. Protik et al. [31] showed that a larger PTV can handle a larger tumour motion while maintaining the same dose homogeneity of the PTV matching the gamma pass rate results reported here.

Still, larger tumour motion amplitudes pose higher demands on intra-fractional motion monitoring and might benefit essentially from motion mitigation techniques or motion compression. Recent studies presented the effectiveness of motion compression for pancreas patients as applied in this study with a standard thermoplastic material [32]. For larger tumour motion amplitudes, as shown for NSCLC patients, the impact on target coverage could be reduced by a combination of gating and volumetric rescanning [33]. The clinical implementation of gating could essentially benefit from internal-external motion correlation, which could be based on different imaging techniques depending on the investigated tumour site. Although gating and rescanning are applied clinically in some centres, the widespread implementation requires improved real-time motion monitoring [21,34,35]. Most recent approaches to improve or replace classical motion mitigation techniques include the reduction of the number of pencil beam spots during rescanning, breathing-phase correlated plan libraries or even enhanced deep inspiration breath hold with oxygen supply [36,37,38,39].

The obtained results showed that the patient’s position (straight or rotated) and as such the beam incidence directions do not systematically influence the motion sensitivity for pancreas treatments with protons, i.e., no impact on the 4DDT was observed. It could also be proven that for the included patient collective 2 to 4 vertical and horizontal beam directions were sufficient to smear out strong motion effects. These findings are especially relevant for the inclusion of more pronounced movements as well as the transition towards carbon ions, where beam angles are always limited and rescanning, therefore, becomes more relevant again, also for small motion amplitudes.

## 5. Conclusions

The hypofractionated proton treatment for pancreas patients based on robust treatment plan optimisation and simple beam configurations showed to be robust against intra-fractional movements up to 3.7 mm being consistent with results from recent phantom measurements [10]. While the patients orientation during immobilisation did not influence the robustness, PTV size and amplitude need to be considered. Still, there was a high inter-patient variability strongly supporting continuous 4DDT calculations in clinical practice. Within this study, necessary tools to investigate the behaviour of carbon ions for the irradiation of pancreatic tumours with a pulsed synchrotron spot scanning beam were developed.

## Figures and Tables

**Table 1:** List of treatment plans included in the analysis; number of treatment plans and patient’s orientation during imaging and treatment (s = straight, r = rotated); pan6, pan13(r) and pan16(r) were acquired in head-first supine position, elsewise head-first-prone (HFP) was applied; the number of fractions, dose per fraction, source from where the breathing period was extracted, mean dose rate over all fractions (DR) [GNP/s] and standard deviation and the inclusion of rescanning; simulated treatment plans are indicated in the last row. * = One treatment plan could not be evaluated due to a large number of spots that was not supported in this version of the 4DDT tool.

Patients	Plans	No. of	Dose/	Breathing Pattern	DR	Rescanning	Simulated
	(s/r)	Fractions	Fraction	Extracted	[GNP/s]	Applied	Non-Rescanned
		(Days)	[Gy(RBE)]	from	(Mean ± Std)		Fractions
pan2	1 (s)	6 (3)	4.0	4DCT	0.52 ± 0.08	yes	3
	2 (r)	4 (2)		4DCT	0.67 ± 0.06	yes	2
pan3	1 (s)	10 (5)	3.75	4DCT	0.60 ± 0.04	yes	5
pan4	1 (s)	6 (3)	3.75	4DCT	0.54 ± 0.05	yes	3
	2 (r)	4 (2)		4DCT	0.54 ± 0.06	yes	2
pan6	1 (s)	10 (5)	3.75	4DCT	0.60 ± 0.03	yes	5
pan7	1 (s)	3 (3)	7.5	4DCT	0.47 ± 0.02	no	-
	2 (r)	4 (2)	3.75	4DCT	0.46 ± 0.03	yes	2
pan8	1 (s)	10 (5)	4.0	4DCT	0.52 ± 0.02	yes	5
pan10	1 (s)	5 (5)	7.5	4DCT	0.51 ± 0.02	no	-
pan11	1 (r)	5 (5)	8.0	4DCT	0.82 ± 0.04	no	-
pan12	1 (s)	4 (2)	3.5	surface scanner	0.96 ± 0.10	yes	-
	2 (r)	6 (3)		4DCT	0.93 ± 0.10	yes	-
pan13	1 (s)	3 (3)	7.5	4DCT	0.98 ± 0.03	no	-
	2 (r)	4 (2)	3.75	surface scanner	1.15 ± 0.01	yes	-
pan15	1 (s)	5 (5)	7.5	surface scanner	0.80 ± 0.03	no	-
pan16	1 (r) *	4 (2)	4.0	surface scanner	0.80 ± 0.05	yes	-
	2 (s)	6 (3)	4.0	4DCT	0.75 ± 0.03	yes	-

**Table 2:** gamma analysis with a 2%/2 mm criteria for the different patient plans with a 20% lower dose cutoff and for the initial PTV region only with the corresponding breathing amplitudes and period extracted from the 4DCT. The orientation of the CT images is indicated behind the plan numbers, namely s for a straight positioning, and r when a rotation was applied.

Patient	Plan	20%	PTV	Breathing	Breathing
	(Orientation)	Isodose	Only	Amplitude [mm]	Period [s]
pan2	1 (s)	79.9	77.4	3.3	4.3
	2 (r)	87.1	91.1	0.4	4.3
pan3	1 (s)	88.7	81.4	2.6	4.0
pan4	1 (s)	93.1	94.6	0.5	3.4
	2 (r)	98.8	98.5	0.3	3.4
pan6	1 (s)	96.0	95.0	0.3	4.2
pan7	1 (r)	93.2	95.0	0.8	4.6
	2 (s)	76.0	74.0	1.7	4.5
pan8	1 (s)	95.4	96.2	1.6	3.8
pan10	1 (s)	95.3	90.8	2.3	4.4
pan11	1 (r)	88.7	89.9	0.4	2.6
pan12	1 (r)	75.2	74.4	0.6	4.2
	2 (s)	97.6	99.6	0.7	3.4
pan13	1 (s)	88.5	93.1	1.3	3.9
	2 (r)	71.6	76.8	1.5	3.9
pan15	1 (s)	91.0	93.1	0.6	4.5
pan16	2 (s)	79.8	88.1	3.7	5.3

## Data Availability

Not applicable.

## Appendix A

**Table A1:** List of DVH parameters from the static treatment plan and resulting from the 4DDT calculation for the target ($D_{98\%}$) and the organs at risk ($D_{2\%}$).

		$D_{98\%}$ [Gy(RBE)]				$D_{2\%}$ [Gy(RBE)]							
		**CTV**		**PTV**		**Liver**		**Kidney**				**Spinal Cord**	
								**Contra**		**Ipsi**			
**Patients**	**Plans**	**Static**	**4DDT**	**Static**	**4DDT**	**Static**	**4DDT**	**Static**	**4DDT**	**Static**	**4DDT**	**Static**	**4DDT**
pan2	1 (s)	14.6	13.8	14.5	13.6	1.2	1.3	0.1	0.1	14.0	14.1	9.28	9.3
	2 (r)	9.8	9.8	9.6	9.6	5.6	5.5	3.8	3.8	10.0	10.1	2.2	2.2
pan3	1 (s)	24.8	24.6	24.4	24.3	12.4	12.2	5.8	6.2	13.8	13.2	12.2	12.2
pan4	1 (s)	14.9	14.8	9.9	10.0	8.3	8.3	0.2	0.1	6.9	7.0	10.1	10.0
	2 (r)	9.8	9.8	8.1	8.1	0.4	0.4	2.8	2.8	6.4	6.4	6.6	6.5
pan6	1 (s)	23.3	23.2	15.9	15.8	14.7	14.8	0.1	0.1	3.2	3.2	0.0	0.0
pan7	1 (s)	14.3	14.6	8.5	8.8	0.2	0.2	8.3	8.0	15.0	15.0	11.7	11.7
	2 (r)	9.3	8.78	2.3	2.6	0.5	0.4	0.9	1.4	10.2	10.0	11.5	11.9
pan8	1 (s)	25.1	25.1	24.4	24.2	29.8	30.6	22.1	21.7	24.3	23.5	13.8	13.8
pan10	1 (s)	24.9	24.8	21.6	22.7	16.1	16.1	5.6	5.7	17.8	17.6	12.1	12.3
pan11	1 (r)	25.3	24.6	18.8	18.8	25.3	25.1	8.2	7.8	15.2	15.8	13.6	13.7
pan12	1 (s)	9.8	9.7	9.6	9.3	5.5	5.2	4.2	4.0	8.0	8.2	5.6	5.6
	2 (r)	14.7	14.7	13.9	13.9	9.8	9.7	4.2	4.1	4.9	4.9	11.1	11.1
pan13	1 (s)	15.1	15.1	13.4	13.8	21.5	21.2	3.8	3.8	21.6	21.6	5.7	5.7
	2 (r)	10.0	8.6	7.7	5.0	13.4	13.5	0.4	0.4	12.5	11.3	0.0	0.0
pan15	1 (s)	24.5	24.2	19.6	20.2	24.6	24.5	6.2	6.2	14.5	14.8	10.7	11.2
pan16	2 (s)	15.0	15.0	13.8	12.9	21.8	21.4	0.0	0.0	9.8	9.0	10.8	11.2

## References

[1] Malouff T.D., Krishnan S., Hallemeier C.L., Haddock M.G., Hoppe B.S., Beltran C., Mahajan A., Trifiletti D. (2020). Carbon Ion Radiotherapy in the Treatment of Pancreatic Cancer: A Review. Pancreas.

[2] Paganetti H. (2014). Relative biological effectiveness (RBE) values for proton beam therapy. Variations as a function of biological endpoint, dose, and linear energy transfer. Phys. Med. Biol..

[3] Batista V., Richter D., Chaudhri N., Naumann P., Herfarth K., Jäkel O. (2018). Significance of intra-fractional motion for pancreatic patients treated with charged particles. Radiat. Oncol..

[4] Bert C., Durante M. (2011). Motion in radiotherapy: Particle therapy. Phys. Med. Biol..

[5] Chang J.Y., Zhang X., Knopf A., Li H., Mori S., Dong L., Lu H.M., Liu W., Badiyan S.N., Both S. (2017). Consensus Guidelines for Implementing Pencil-Beam Scanning Proton Therapy for Thoracic Malignancies on Behalf of the PTCOG Thoracic and Lymphoma Subcommittee. Int. J. Radiat. Oncol. Biol. Phys..

[6] Edvardsson A., Nordström F., Ceberg C., Ceberg S. (2018). Motion induced interplay effects for VMAT radiotherapy. Phys. Med. Biol..

[7] Pakela J.M., Knopf A., Dong L., Rucinski A., Zou W. (2022). Management of Motion and Anatomical Variations in Charged Particle Therapy: Past, Present, and Into the Future. Front. Oncol..

[8] Kostiukhina N., Georg D., Rollet S., Kuess P., Sipaj A., Andrzejewski P., Furtado H., Rausch I., Lechner W., Steiner E. (2017). Advanced Radiation DOSimetry phantom (ARDOS): A versatile breathing phantom for four dimensional (4D) radiation therapy and medical imaging. Phys. Med. Biol..

[9] Kostiukhina N., Palmans H., Stock M., Georg D., Knäusl B. (2019). Dynamic lung phantom commissioning for end-to-end 4D dose assessment in proton therapy. Phys. Med. Biol..

[10] Lebbink F., Stock M., Georg D., Knäusl B. (2022). The Influence of Motion on the Delivery Accuracy When Comparing Actively Scanned Carbon Ions versus Protons at a Synchrotron-Based Radiotherapy Facility. Cancers.

[11] Pfeiler T., Bäumer C., Engwall E., Geismar D., Spaan B., Timmermann B. (2018). Experimental validation of a 4D dose calculation routine for pencil beam scanning proton therapy. Z. Für Med. Phys..

[12] Spautz S., Jakobi A., Meijers A., Peters N., Löck S., Knopf A.C., Troost E.G., Richter C., Stützer K. (2022). Experimental validation of 4D log file-based proton dose reconstruction for interplay assessment considering amplitude-sorted 4DCTs. Med. Phys..

[13] Kostiukhina N., Palmans H., Stock M., Knopf A., Georg D., Knäusl B. (2020). Time-resolved dosimetry for validation of 4D dose calculation in PBS proton therapy. Phys. Med. Biol..

[14] Zechner A., Ziegler I., Hug E., Lütgendorf-Caucig C., Stock M. (2022). Evaluation of the inter- and intrafraction displacement for head patients treated at the particle therapy centre MedAustron based on the comparison of different commercial immobilisation devices. Z. Fur Med. Phys..

[15] Stock M., Georg D., Ableitinger A., Zechner A., Utz A., Mumot M., Kragl G., Hopfgartner J., Gora J., Böhlen T. (2018). The technological basis for adaptive ion beam therapy at MedAustron: Status and outlook. Z. Fur Med. Phys..

[16] Weistrand O., Svensson S. (2015). The ANACONDA algorithm for deformable image registration in radiotherapy. Med. Phys..

[17] ICRU83 (2010). Special Considerations Regarding Absorbed-Dose and Dose–Volume Prescribing and Reporting in IMRT. J. ICRU.

[18] Pan C.C., Kavanagh B.D., Dawson L.A., Li X.A., Das S.K., Miften M., Ten Haken R.K. (2010). Radiation-associated liver injury. Int. J. Radiat. Oncol. Biol. Phys..

[19] Mori S., Asakura H., Kandatsu S., Kumagai M., Baba M., Endo M. (2008). Magnitude of Residual Internal Anatomy Motion on Heavy Charged Particle Dose Distribution in Respiratory Gated Lung Therapy. Int. J. Radiat. Oncol. Biol. Phys..

[20] Trnková P., Knäusl B., Actis O., Bert C., Biegun A.K., Boehlen T.T., Furtado H., McClelland J., Mori S., Rinaldi I. (2019). Clinical implementations of 4D pencil beam scanned particle therapy: Report on the 4D treatment planning workshop 2016 and 2017. Phys. Med..

[21] Zhang Y., Trnkova P., Toshito T., Heijmen B., Richter C., Aznar M., Albertini F., Bolsi A., Daartz J., Bertholet J. (2023). A survey of practice patterns for real-time intrafractional motion-management in particle therapy. Phys. Imaging Radiat. Oncol..

[22] Ribeiro C.O., Knopf A., Langendijk J.A., Weber D.C., Lomax A.J., Zhang Y. (2018). Assessment of dosimetric errors induced by DIR methods in 4D pencil beam scanned proton treatment planning for liver tumours. Radiother. Oncol..

[23] Nenoff L., Ribeiro C.O., Matter M., Hafner L., Josipovic M., Langendijk J.A., Persson G.F., Walser M., Weber D.C., Lomax A.J. (2020). DIR uncertainty for inter-fractional dose accumulation of lung cancer proton therapy. Radiother. Oncol..

[24] Amstutz F., Nenoff L., Albertini F., Ribeiro C.O., Knopf A.C., Unkelbach J., Weber D.C., Lomax A.J., Zhang Y. (2021). An approach for estimating dosimetric uncertainties in deformable dose accumulation in pencil beam scanning proton therapy for lung cancer. Phys. Med. Biol..

[25] Dolde K., Naumann P., Dávid C., Gnirs R., Kachelrieß M., Lomax A.J., Saito N., Weber D.C., Pfaffenberger A., Zhang Y. (2018). 4D dose calculation for pencil beam scanning proton therapy of pancreatic cancer using repeated 4DMRI datasets. Phys. Med. Biol..

[26] Duetschler A., Prendi J., Safai S., Weber D.C., Lomax A.J., Zhang Y. (2023). Limitations of phase-sorting based pencil beam scanned 4D proton dose calculations under irregular motion. Phys. Med. Biol..

[27] Engwall E., Glimelius L., Hynning E. (2018). Effectiveness of different rescanning techniques for scanned proton radiotherapy in lung cancer patients. Phys. Med. Biol..

[28] Ribeiro C.O., Visser S., Korevaar E.W., Sijtsema N.M., Anakotta R.M., Dieters M., Both S., Langendijk J.A., Wijsman R., Muijs C.T. (2021). Towards the clinical implementation of intensity-modulated proton therapy for thoracic indications with moderate motion: Robust optimised plan evaluation by means of patient and machine specific information. Radiother. Oncol..

[29] Meijers A., Knopf A.C., Crijns A.P., Ubbels J.F., Niezink A.G., Langendijk J.A., Wijsman R., Both S. (2020). Evaluation of interplay and organ motion effects by means of 4D dose reconstruction and accumulation. Radiother. Oncol..

[30] Lim P.S., Pica A., Hrbacek J., Bachtiary B., Walser M., Lomax A.J., Weber D.C. (2020). Pencil Beam Scanning Proton Therapy for Paediatric Neuroblastoma with Motion Mitigation Strategy for Moving Target Volumes. Clin. Oncol..

[31] Protik A., Herk M.V., Witte M., Sonke J.J. (2017). The impact of breathing amplitude on dose homogeneity in intensity modulated proton therapy. Phys. Imaging Radiat. Oncol..

[32] Schneider S., Stefanowicz S., Jentsch C., Lohaus F., Thiele J., Haak D., Valentini C., Platzek I., Troost E.G.C., Hoffmann A.L. (2023). Reduction of intrafraction pancreas motion using an abdominal corset compatible with proton therapy and MRI. Clin. Transl. Radiat. Oncol..

[33] Köthe A., John A., Chiara A., Safai S., Bizzocchi N., Roelofs E., Even A.J.G., Charles D., Fattori G. (2022). The impact of organ motion and the appliance of mitigation strategies on the effectiveness of hypoxia-guided proton therapy for non-small cell lung cancer. Radiother. Oncol..

[34] Gulyas I., Trnkova P., Knäusl B., Widder J., Georg D., Renner A. (2022). A novel bone suppression algorithm in intensity-based 2D/3D image registration for real-time tumour motion monitoring: Development and phantom-based validation. Med. Phys..

[35] Nankali S., Worm E.S., Thomsen J.B., Stick L.B., Bertholet J., Høyer M., Weber B., Mortensen H.R., Poulsen P.R. (2023). Intrafraction tumor motion monitoring and dose reconstruction for liver pencil beam scanning proton therapy. Front. Oncol..

[36] Maradia V., Water S.V.D., Meer D., Weber D.C., Lomax A.J., Psoroulas S. (2022). Ultra-fast pencil beam scanning proton therapy for locally advanced non-small-cell lung cancers: Field delivery within a single breath-hold. Radiother. Oncol..

[37] Bertschi S., Krieger M., Weber D.C., Lomax A.J., van de Water S. (2022). Impact of spot reduction on the effectiveness of rescanning in pencil beam scanned proton therapy for mobile tumours. Phys. Med. Biol..

[38] Hamaide V., Souris K., Dasnoy D., Glineur F., Macq B. (2023). Real-time image-guided treatment of mobile tumors in proton therapy by a library of treatment plans: A simulation study. Med. Phys..

[39] Emert F., Missimer J., Eichenberger P.A., Walser M., Gmür C., Lomax A.J., Weber D.C., Spengler C.M. (2021). Enhanced Deep-Inspiration Breath Hold Superior to High-Frequency Percussive Ventilation for Respiratory Motion Mitigation: A Assessment Toward Optimized Lung Cancer Treatment With Proton Therapy. Front. Oncol..

